# Toward the conservation of the endemic monotypic fish genus *Aulopyge* from the Balkan Dinaric karst: Integrative assessment of introduced and natural population

**DOI:** 10.1002/ece3.7108

**Published:** 2020-12-10

**Authors:** Jasmina Ludoški, Ljubinka Francuski, Milica Lukač, Radoslav Dekić, Vesna Milankov

**Affiliations:** ^1^ Department of Biology and Ecology Faculty of Sciences University of Novi Sad Novi Sad Serbia; ^2^ Groningen Institute for Evolutionary Life Sciences University of Groningen Groningen the Netherlands; ^3^ Faculty of Sciences University of Banja Luka Banja Luka Bosnia and Herzegovina

**Keywords:** *COI* mtDNA, *cyt b* mtDNA, DNA barcode database, endangered cyprinid, geometric morphometrics, linear morphometrics

## Abstract

The complex biogeographical history of the Balkan Peninsula caused remarkable freshwater fish diversity and endemism, among which Cyprinidae fish dominate. The Dinaric karst was a Pleistocene refugium and it harbors ancient and endemic cyprinids, including *Aulopyge huegelii*, a sole representative of its genus. Being highly distributionally restricted, it faces various threats that promote a critical decline in population abundance and even population extinction. Phenotypic and molecular diversity of the introduced (Šator Lake, Šator Mountain) and natural (Studena River, Duvanjsko Polje) populations of Dalmatian barbelgudgeon from Bosnia and Herzegovina was studied by using two mitochondrial genes and morphometric traits (linear and geometric morphometrics). Nonparametric ANOVA showed that two analyzed populations significantly differed in six linear measurements, except snout length and postorbital head length. Contrary to centroid size, two populations were found to be significantly different in body shape. Deformation grids indicated that individuals from Studena River are characterized by wider and slightly shorter body comparing to individuals from Šator Lake. Incongruence in cytochrome *c* oxidase subunit I (*COI*) and cytochrome *b* (*cyt b*) mitochondrial DNA (mtDNA) variation was observed since a common *COI* haplotype was observed, while four and three *cyt b* haplotypes were registered in Šator Lake and Studena River, respectively. Since it was demonstrated that *cyt b* mtDNA was a faster evolving gene, we encourage its use in intraspecies studies, especially for evaluating the connectivity of fragmented populations and for studying the evolutionary footprint of the processes incorporated into the distinctive evolution of *Aulopyge*. Finally, findings herewith provide a firm basis for designing a long‐term sustainable conservation strategy for endemic species in Dinaric karst.

## Graphical abstract

The manuscript deals with the issue of phenotypic and molecular diversity of the introduced (Šator Lake, Livanjsko Polje) and natural (Studena River, Duvanjsko Polje) populations of Dalmatian barbelgudgeon from Bosnia and Herzegovina based on the use of two mitochondrial genes and morphometric traits (linear and geometric morphometrics). Given that the Dinaric karst was a Pleistocene refugium and it harbors ancient and endemic cyprinids, including *Aulopyge huegelii*, our results provide deeper insights into the freshwater fish biodiversity of one of the most famous karstic regions of the world.

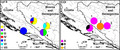

## 1 INTRODUCTION

**FIGURE 1:**
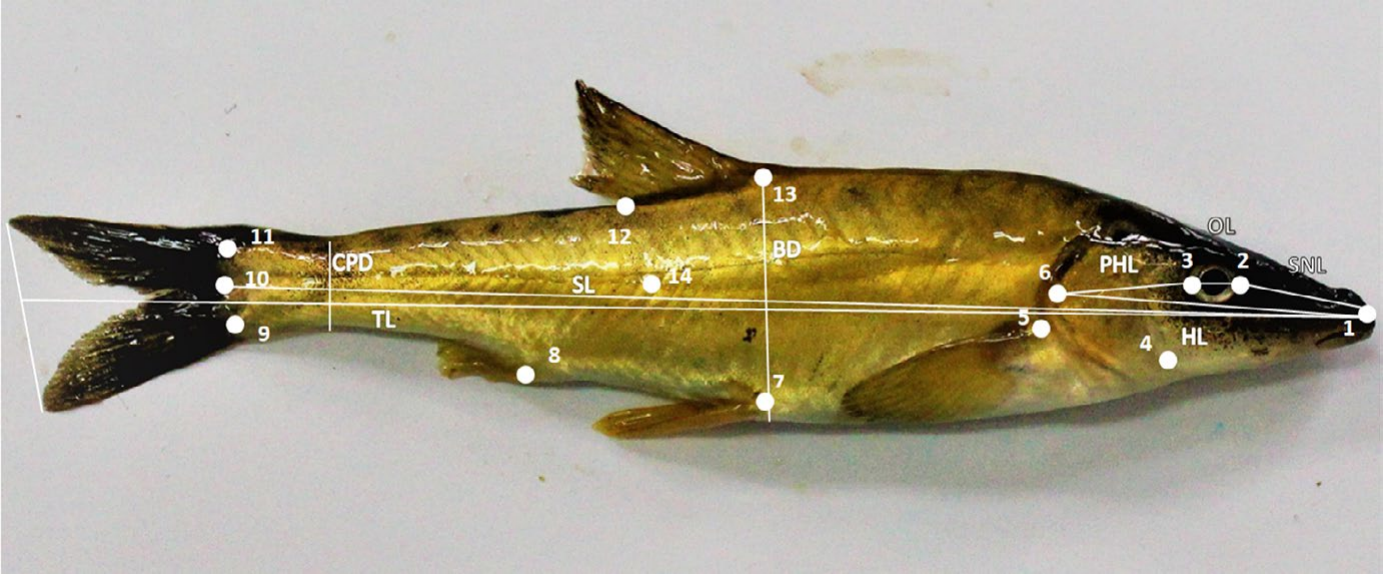
Linear morphometric measurements: BD, body depth; CPD, caudal‐peduncle depth; HL, head length; OL, orbit length; PHL, postorbital head length; SL, standard length; SNL, snout length; TL, total length. The white dots represent 13 homologous landmarks used for geometric morphometry (landmarks 1–13). The dot marked with number 14 is used only for the “unbend” procedure

*Aulopyge huegelii* Heckel, 1843, the Dalmatian barbelgudgeon (Figure 1), solely represents a monotypic genus (Howes, 1987) of great conservation and systematics concern. Within the family Cyprinidae, the phylogeny of Cyprininae subfamily has long been in focus of discussion due to controversial relationships among European *Barbus* sensu *stricto*, *Aulopyge*, *Capoeta*, African *Barbus* sensu *lato*, and Asian Cyprininae fishes (e.g., Wang et al., 2013). Indeed, the genus *Barbus* is regarded as a polyphyletic group since some *Barbus* species are more closely related to *A. huegelii* than to other barbels (Howes, 1987; Machordom & Doadrio, 2001). It was suggested that the common tetraploid ancestor was shared by European *Barbus* sensu *stricto*, including *Aulopyge*, and Asian *Cyprinion* and *Scaphiodonichthys* (Benovics et al., 2017; Collares‐Pereira, 1994; Howes, 1987; Machordom & Doadrio, 2001; Wang et al., 2013) in the Qinghai‐Tibetan Plateau (QTP) about 19 MYR (Benovics et al., 2017). The orogenesis of the QTP was tightly linked with the radiation of Cyprininae (29‐18 MYR) and their first migration route, which was followed by the divergence of European *Barbus* sensu *stricto* (Palaearctic tetraploids) and the tetraploid *Aulopyge* lineage (around 16.6‐15.5 MYR) (Machordom & Doadrio, 2001; Tsigenopoulos & Berrebi, 2000; Wang et al., 2013). As a result of ancient origin, *Aulopyge* is a basal lineage of *Barbus* sensu *stricto* and the sister group of *Barbus* and *Luciobarbus* subgenera (Machordom & Doadrio, 2001). The next migration wave of cyprinids from Asia to the peri‐Mediterranean region occurred during Plio‐Pleistocene, and it was associated with complex geomorphological and hydrogeological events that lead to further diversification and biogeographical structuring of barbels (Wang et al., 2013).

Intensive biogeographical history of the Balkan Peninsula caused remarkable freshwater fish diversity and endemism, among which Cyprinidae dominate (Oikonomou et al., 2014). The Dinaric karst, as the area of karstic springs, caves, and subterranean hydrological network, was a Pleistocene refugium and it harbors ancient and endemic cyprinids, including *A. huegelii* (Oikonomou et al., 2014). *Aulopyge huegelii* possess a unique morphological assemblage that includes autapomorphic and synapomorphic traits with other cyprinids (Bless & Riehl, 2002; Howes, 1987). Regarding its phylogenetic relictness, evolutionary uniqueness, and endemism, Dalmatian barbelgudgeon is considered as a species of high priority in conservation of the notable, but sensitive and thus vulnerable, Dinaric karst region. However, population structure of the species is still poorly understood, which implies further research related to spatial and temporal distribution of molecular and phenotypic variation. This is of fundamental importance since Dalmatian barbelgudgeon is seriously threatened due to habitat degradation and loss, water pollution, unsustainable water extraction, and introduction of alien invasive species (Crivelli, 2006). Hence, *A. huegelii* faces various threats, leading its populations to critically small abundance or even extinction (http://www.iucnredlist.org/details/61350/0). Thus, the species is assessed as Endangered B1ab(iii,v) according to IUCN (http://www.iucnredlist.org/details/61350/0). As a consequence, urgent habitat protection and population monitoring of the species were proposed (Ćaleta et al., 2009). Moreover, *A. huegelii* fulfilled both criteria for assessing biodiversity conservation priorities ‐ endemism and phylogenetic diversity as the measures of the amounts of evolutionary history (Isaac et al., 2007). More importantly, deeper insights into genetics of the species might uncover ancient history of Cyprininae and confirm the Oriental‐ to‐Palaearctic migration route of freshwater fish.

Phylogenetic uniqueness of the Dalmatian barbelgudgeon is associated with its highly restricted distribution limited to the Dinaric karst of Bosnia and Herzegovina (small rivers in Livanjsko Polje, Glamočko Polje and Duvanjsko Polje, Lakes Blidinje and Buško) and Croatia (Krka. Cetina, Čikola, Ruda and Rumin Rivers) (Ćaleta et al., 2015, 2019; Mrakovčić & Mišetić, 1990; Vuković & Ivanović, 1971). Livanjsko Polje is the largest karst field in the world (Ritter‐Studnićka & Grgić, 1971), characterized by extraordinary karstic features circled by mountain ranges, including Šator Mt. One of the geomorphological and hydrological phenomena of this karstic region is a glacial Šator Lake situated on the Šator Mt. Šator Lake has been noted as one of two localities (another one being Blidinje Lake) where Dalmatian barbelgudgeon was introduced (Delić et al., 2005). Unlike natural habitats of the species that belong to the Adriatic basin, Šator Lake belongs to the Black Sea basin through the Una and Danube rivers. Delić et al. (2005) regarded Šator Lake as a locality at the highest altitude of *Aulopyge* populations. Consequently, the translocated population of the unknown origin faces harsh environment which favors specific adaptations (Delić et al., 2005). In addition, unlike other populations, *Aulopyge* from Šator Lake is supposed to spend its whole life cycle in aboveground water (Delić et al., 2005). Another large Dinaric karst field, Duvanjsko Polje located in Southeastern part of Bosnia Herzegovina is a 20 km long and 12 km wide. Duvanjsko Polje represented by paleogenic limestones and dolomites from Jurassic and Cretaceous, while newer sediments origin from Miocene (Radoš et al., 2012). One of the geological unique features is the subterranean Studena River that flows through Duvanjsko Polje, while in southwestern corner of the field the river goes underground within the main estaville and further continuing through karstic subterranean network. Then, the Studena River partially re‐emerges at the source of Ricina and finally empties into the reservoir of Buško Blato (Radoš et al., 2012). Hence, Studena River is a natural home of the Dalmatian barbelgudgeon that faces both underground and belowground environments.

Therefore, the primary objective of our study was to evaluate intra‐ and interpopulation phenotypic and molecular diversity of the Dalmatian barbelgudgeon in order to provide invaluable data for conservation management of this unique evolutionary cyprinids clade. In this study, we complementary used phenotypic traits (linear and geometric morphometrics) and two mitochondrial genes (cytochrome *c* oxidase I ‐ *COI* and cytochrome *b* – *cyt b*) of the two populations, one of them was recently introduced in Šator Lake (Šator Mt), while the second was autochtonous population from Studena River (Duvanjsko Polje). The data on variation of the two mitochondrial genes was contrasting to evaluate their usefulness in conservation and forensics of this endangered fish species. Indeed, choice of appropriate molecular marker is a prerequisite for population discrimination and identification of divergent units. Given the power of DNA taxonomy in the biodiversity assessment, our additional goal was to fill the gap of DNA barcode database of the vulnerable ichthyofauna in the Mediterranean Hotspot region. Furthermore, regarding that phenotypic variation of *A. huegelli* has been studied based on linear morphometrics (Dekić et al., 2016; Mihinjač, 2018; Mušović et al., 2018) we choose to contrast traditional approach (linear measurements) with geometric morphometrics (body size and shape). Hence, study of the two populations, one faces to different selection regimes of under‐ and aboveground ecosystems (a sinking Studena River) and another population adapted to aboveground environment (Šator Lake), further provides comprehensive information on the phenotypic variation. It is important from the ecological perspective since pattern of subtle intraspecific phenotypic (e.g., body shape) is tightly linked to environmental variables (Collin & Fumagalli, 2011). Finally, findings herewith further contribute to understanding cryptic diversity on the Balkan Peninsula and provide a firm basis for designing a long‐term sustainable conservation strategy for endemic species in Dinaric karst.

## 2 MATERIAL AND METHODS

### 2.1 Sample collection

**FIGURE 2:**
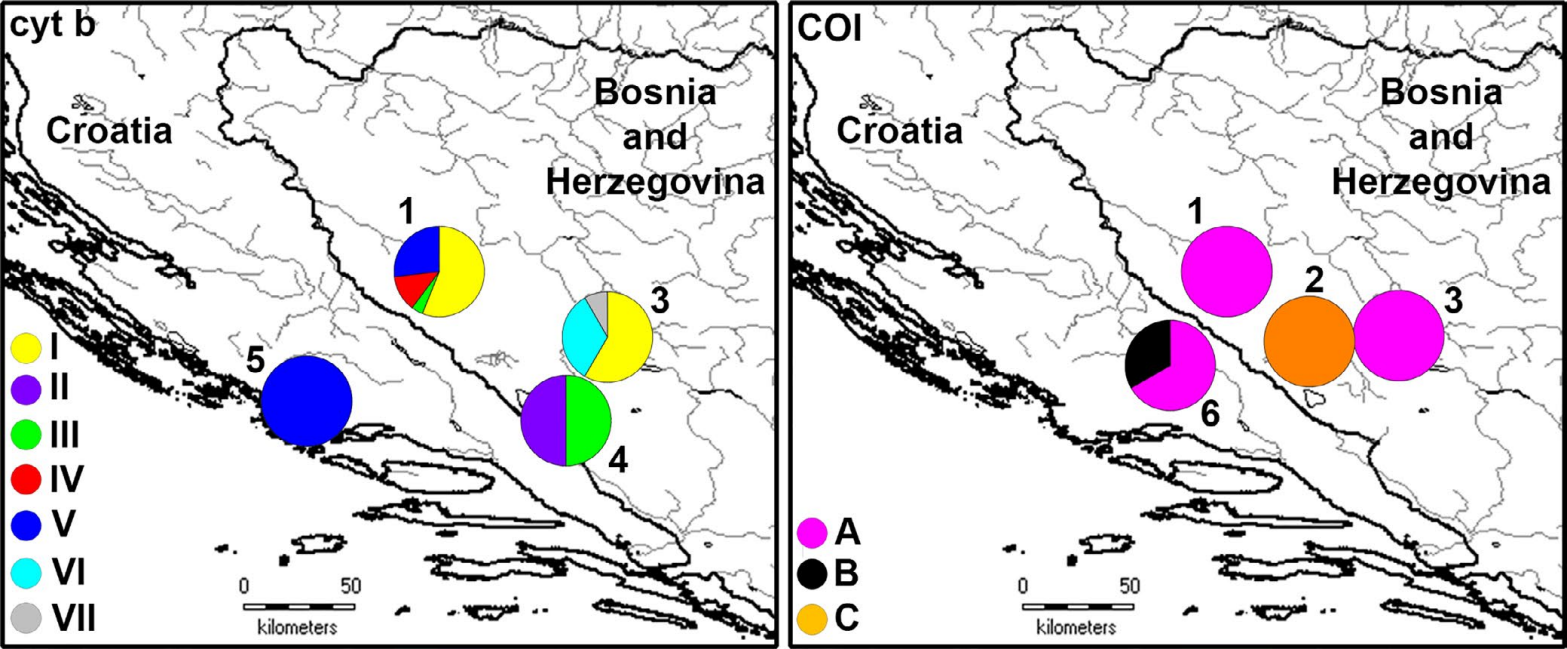
Map of the studied area. Bosnia and Herzegovina: 1. Šator Lake; 2. Livno drainage; 3. Studena River; 4. Buško Lake; Croatia: 5. Krka River and 6. Cetina River. *Cyt b* (I‐VII) and COI (A‐C) mtDNA haplotype distribution is presented, where the size of each pie slice represents the number of individuals with that haplotype

Fish were collected from two localities in Bosnia and Herzegovina: Šator Lake (1,488 m a.s.l.; 44°9′54.54″N, 16°36′5.54″E) and Studena River in Duvanjsko Polje (1,050 m a.s.l.; 43°40′34.09″N, 17°11′13.01″E) using sport fishing technique with a fishing net (“čerenac”). Sampling was conducted in 2015 in Šator Lake (25 individuals) by R. Dekić and M. Lukač, and in 2017 in Studena River (13 individuals) by J. Pavličević. Species determination was carried out using morphological keys (Kottelat & Freyhof, 2007; Vuković & Ivanović, 1971). During the sampling of fish in Šator Lake, water parameters (water temperature, pH, dissolved oxygen, oxygen saturation, conductivity, and turbidity) at five points were also collected (Table S1). Sampling was regulated by Permission for ichthyofauna research (o3‐3‐24/3‐182/18, 27.4.2018) issued by the Ministry of Agriculture, Water Management and Forestry of the Federation of Bosnia and Herzegovina. Morphometric analyses were performed on 38 individuals, while for the analyses of mtDNA genes 36 and 33 individuals were used‐ *cyt b* mtDNA: 24 from Šator Lake and 12 from Studena River; *COI* mtDNA: 23 from Šator Lake and 10 from Studena River. In addition, to study *cyt b* mtDNA and *COI* mtDNA variability, three *cyt b* mtDNA sequences (two from Buško Lake in Bosnia and Herzegovina and one from Krka River in Croatia) and four *COI* mtDNA sequences (one from Livno drainage in Bosnia and Herzegovina and three from Cetina River in Croatia) were downloaded from GenBank (Figure 2; Table S2). Therefore, the total sample size included 39 *cyt b* mtDNA and 37 *COI* mtDNA sequences. Due to the lack of sexual dimorphism revealed by preliminary analysis, female and male specimens were not considered separately.

### 2.2 Morphometric analyses

A total of 38 specimens from Šator Lake (25 individuals) and Studena River (13 individuals) were analyzed. Digital images of the right lateral side were photographed with a Nikon D7100 digital camera with 50‐mm f/1.4D objective appointed on a tripod stand and positioned vertical to the surface of the object. Additionally, since the linear measurements and precise position for landmarks could not have been precisely determined on right‐side images, for six specimens left lateral side was captured and use in morphometric analyses. The scale factor is determined for each photo according to the millimeter scale before the digitization process.

#### 2.2.1 Linear measurements

Linear morphometric characteristics were measured (in mm) on digital images using measurement mode in tpsDig 2.30 (Rohlf, 2017). According to the standard for cyprinoid fishes (Armbruster, 2012), the total of eight linear measurements used for analysis were as follows: total length (TL), standard length (SL), orbit length (OL), snout length (SNL), postorbital head length (PHL), head length (HL), body depth (BD), and caudal‐peduncle depth (CPD) (Figure 1). In order to remove allometry effect, linear measurements were transformed into (a) the percentage ratios of measured distance and SL (for OL, SNL, HL, BD, and CPD) and HL (for OL, SNL, and PHL), and (b) size‐independent adjusted measurements. The second one was calculated in a way the size component was eliminated from a multivariate data set of measured distances following Elliot et al. (1995) procedure using formula:

$$M_{\text{adj}}=M\;{\left(L_{s}/L_{o}\right)}^{b}$$

where *M* is the original morphometric measurement, *M*_adj_ the size‐independent measurement, *L*_s_ the overall mean of SL for all fish in both population, *L*_o_ the SL of the fish, *b* the slope (allometric coefficient) of regression of log*M* on log*L*_o_ calculated for both population. Correlation coefficients between adjusted size‐independent variables and SL were calculated in order to confirm that the size effect was eliminated and significant correlation was not found.

Prior to statistical analysis, a normal distribution test was performed (for each measure separately) on percentage ratios and size‐adjusted measurements data using both Shapiro‐Wilk test (for univariate normality). Also, for matrix of size‐adjusted measurements, Doornik & Hansen omnibus test for multivariate normality was done.

Nonparametric ANOVA (with 10,000 permutation runs) was used to determine differences in variances among two populations. Furthermore, principal component analysis (PCA) based on size‐adjusted measurements was performed. All statistical analysis on linear measures was executed in PAST 4.03 (Hammer et al., 2001) software.

#### 2.2.2 Geometric morphometrics

In total, 13 homologous landmarks (Figure 1) were digitized on the images using tpsDig 2.30 (Rohlf, 2017): 1. snout tip; 2. most anterior point of the eye outline; 3. most posterior point of the eye outline; 4. lower position on preoperculum; 5. superior point of pectoral fin; 6. posterior most edge of operculum; 7. anterior insertion of pelvic fin; 8. anterior point of anal fin; 9. ventral point of caudal fin; 10. posterior point of lateral line; 11. dorsal point of caudal fin; 12. posterior point of dorsal fin; 13. anterior point of dorsal fin. In order to remove effect of unnatural bending of specimens, landmark 14 was added and positioned on the lateral line half‐way between landmarks 6 and 10. Unbending procedure was implemented in tpsUtil 1.74 (Rohlf, 2017) and four landmarks were aligned (1, 6, 10, 14). Landmark (14) was removed from following analysis. Procrustes fit procedure was used for superimposing landmarks' coordinates (Dryden & Mardia, 1998; Klingenberg & McIntyre, 1998) and information about shape variable (Procrustes coordinates) was obtained. Before unbending procedure was applied, centroid size (size variable) was also calculated. To test for the presence of allometry (the relationship between size and shape), a multivariate regression of Procrustes coordinates against centroid size on pooled within‐group (pooled by population) variation was conducted. Permutation test with 10,000 iterations was used for checking significance of the allometry.

To examine body size differences among defined groups, nonparametric ANOVA with 10,000 permutaions was used. In order to determine and visualize differences in body shape between two a priori defined groups (populations), the matrix of shape variables (Procrustes coordinates) was subjected to discriminant function analysis (DFA). The dependability of the discrimination among groups is assessed using leave‐one‐out (jackknifing) cross‐validation procedure. Finally, to estimate the extent of morphological variation of body shape, Procrustes distances between pairs of individuals and morphological disparity were calculated for each population. The morphological disparity was calculated as variance (Procrustes variance; Zelditch et al., 2012) and compared between populations with a permutation test with 1,000 iterations. Statistical analysis for geometric morphometrics was performed using MorphoJ 1.06d (Klingenberg, 2011), PAST 4.03 (Hammer et al., 2001), and DisparityBox6i (Sheets, 2007) software.

#### 2.2.3 Length‐weight relationship (LWR)

To analyze the length‐weight relationship (LWR) of fish body we measured the weight of 11 and 13 individuals from Šator Lake and Studena River, respectively, using digital weighing scale KERN 440‐33 to the nearest 0.01 g. The relationship between standard length (in cm) and weight was estimated according to the formula (Froese, 2006):

$$W=aL^{b}$$

where *W* ‐ weight of fish, *L* ‐ standard length (SL) of fish, *a* ‐ scaling coefficient, *b* ‐ length exponent (shape parameter for the body form). Model of LWR was transformed into linear type of data using logarithmic transformation (natural logarithm):

$$\ln\left(W\right)=\ln\left(a\right)\;+\;b\ln\left(L\right).$$

where ln(*a*) is the intercept and *b* is the slope of linear regression (Le Cren, 1951).

The squared correlation (*r* ^2^) that indicates reliability of regression model fit was calculated for each population separately. The value of parameter *b* shows the type of somatic growth pattern. Isometric growth is represented if *b* = 3 and allometric (positive or negative) if *b* ≠ 3. The *b* value was estimated for each population separately. To assess whether the obtained *b* values statistically differed from the isometric value (*b* = 3), *t* test was used (Froese, 2006). Statistical analysis was performed in Microsoft Excel 2010 and PAST 4.03 (Hammer et al., 2001) software.

### 2.3 Molecular analysis

#### 2.3.1 DNA extraction, PCR amplification, and sequencing

Total genomic DNA from tissue samples was extracted from 36 fish using NucleoSpin^®^ Tissue DNA extraction kit (MACHEREY‐NAGEL) and following the manufacturer's protocol. The tissue and the extracted DNA were stored at −20°C. A fragment of *cyt b* mtDNA was amplified using Glu‐F (5′‐GAAGAACCACCGTTGTTATTCAA‐3′)/Thr‐R (5′‐ACCTCCRATCTYCGGATTACA‐3′) primer pair (Zardoya & Doadrio, 1998), while the amplification of the 5′ end of *COI* mtDNA was done by LCO‐1490 (5′‐G GTCAACAAATCATAAAGATATTGG‐3′)/HCO‐2198 (5′‐TTAAACTTCAGGGTGACCAAAAAATCA‐3′) primer pair (Folmer et al., 1994). PCR reactions were performed using an illustra PuReTaq Ready‐To‐Go PCR Beads kit (GE Healthcare Life Sciences). PCR conditions for *cyt b* mtDNA and *COI* mtDNA amplification were described in Palandačić et al. (2012) and Milankov et al. (2009), respectively. To check the success of reactions, amplification products were separated on a 2% agarose gel. PCR products were then purified using ExoSAP‐IT™ PCR Product Cleanup Reagent (Thermo Fisher Scientific), and bidirectionally sequenced on ABI3730XL by Macrogen.

#### 2.3.2 Data analyses

Chromatograms obtained by mtDNA sequencing were edited in Chromas 2.6 (Tehnelysium Pty Ltd) for erroneously called bases, while sequence alignment was performed in BioEdit 7.2.5 (Hall, 1999). Haplotype networks were constructed in Network 10.1.0.0. (Fluxus Technology Ltd.) using a median joining approach. Haplotype divergences (*p*‐distances) were obtained using MEGA X 10.0.5 (Kumar et al., 2018) by dividing the number of nucleotide differences by the total number of nucleotides compared. In addition, we determined total and private haplotype numbers per geographic sample.

Estimates of *θ* (where *θ* = 2*Nu*, *N* is the effective population size and *u* is the average mutation rate per locus per generation), were based on the infinite‐allele model implemented in Arlequin version 3.11 (Excoffier et al., 2005). Using both mtDNA markers, four *θ* estimates were calculated: *θ_K_* obtained from the distinct number of haplotypes (*K*), *θ_H_* obtained from the observed homozygosity (*H*), *θ_S_* obtained from the observed number of segregating sites (*S* = number of polymorphic sites) and *θ_π_* obtained from the mean number of pairwise differences (*π*). *θ_K_* was estimated from the infinite‐allele mutation model equilibrium relationship between the expected number of haplotypes (*K*), the sample size (*n*) and *θ* using Ewens' sampling formula. *θ_S_* Watterson's estimator is based on the infinite‐site mutation model relationship between the number of segregating sites (*S*), the sample size (*n*) and *θ*. Tajima's estimator of *θ* (*θ_π_*) is also based on the infinite‐site mutation model, but on the relationship between the mean number of pairwise differences (*p*) and *θ*.

To assess the correlation of genetic distance (*p*‐distances obtained from *cytb* mtDNA sequences) and phenotypic distances (Euclidian distance calculated from Procrustes‐fitted landmark coordinates) Mantel's test with 10,000 permutations was applied.

## 3 RESULTS

### 3.1 Linear morphometric analysis

Univariate analysis of normality suggested that linear measures TL, SL, BD, and CPD, morphometric ratio OL(%HL) and all six size‐adjusted measures deviated from the normal distribution (Shapiro‐Wilk test, *p* < .05). Also, multivariate normality test indicated significant deviation from normal distribution for size‐adjusted measurements (Doornik & Hansen omnibus test Ep = 28.43, *p* < .01).

Mean values of all raw linear and size‐independent adjusted measures, and four morphometric ratios [SNL(%HL), PHL(%HL), BD(%SL), CPD(%SL)] were higher in Studena River population than values recorded in Šator Lake population. Furthermore, for all size‐adjusted measures, there is a gap in range values between two populations contrary to raw linear and morphometric ratio measures where ranges largely overlapped (Table 1).

**TABLE 1:** Population mean, minimal and maximal values of morphometric traits of *Aulopyge huegelii*

Linear measures	Šator Lake		Studena River		Nonparametric ANOVA
	Ranges	Mean ± *SD*	Ranges	Mean ± *SD*	
TL (mm)	56.34–126.07	79.23 ± 18.90	95.61–146.65	120.73 ± 14.83	***
SL (mm)	47.66–107.66	66.86 ± 15.56	80.84–124.69	103.12 ± 12.52	***
OL (mm)	3.14–5.52	4.00 ± 0.63	4.03–5.89	5.15 ± 0.52	***
SNL (mm)	4.75–12.84	7.82 ± 2.11	8.15–12.58	10.71 ± 1.45	***
PHL (mm)	5.27–11.91	8.24 ± 1.83	9.13–13.96	11.51 ± 1.20	***
HL (mm)	13.86–29.35	19.63 ± 4.43	21.10–31.62	26.78 ± 3.07	***
BD (mm)	9.82–23.18	13.76 ± 3.71	20.15–30.15	25.02 ± 2.92	***
CPD (mm)	3.67–8.54	5.22 ± 1.27	6.92–11.70	8.74 ± 1.28	***
OL (%SL)	5.07–7.53	6.11 ± 0.70	4.39–5.55	5.02 ± 0.35	***
OL (%HL)	17.40–25.90	20.79 ± 2.49	17.40–21.52	19.29 ± 1.10	*
SNL (%SL)	9.97–12.73	11.61 ± 0.81	9.73–11.31	10.38 ± 0.58	***
SNL (%HL)	34.27–44.04	39.51 ± 2.65	36.47–42.25	39.93 ± 1.50	Ns
PHL (%HL)	38.02–47.05	42.00 ± 1.88	40.46–45.32	43.06 ± 1.49	Ns
HL (%SL)	26.07–33.11	29.41 ± 1.48	24.57–28.23	26.00 ± 1.04	***
BD (%SL)	18.39–23.51	20.45 ± 1.41	21.82–26.23	24.30 ± 1.24	***
CPD (%SL)	6.76–8.73	7.82 ± 0.55	7.55–9.38	8.47 ± 0.48	**
OL_adj_	3.54–4.46	4.03 ± 0.28	4.64–5.56	5.16 ± 0.30	***
SNL_adj_	6.93–8.70	7.79 ± 0.47	10.07–11.71	10.71 ± 0.60	***
PHL_adj_	7.29–9.93	8.25 ± −0.54	10.62–12.08	11.52 ± 0.46	***
HL_adj_	17.32–21.98	19.65 ± 0.98	25.10–28.88	26.79 ± 1.03	***
BD_adj_	12.23–16.14	13.71 ± 0.88	22.74–26.98	25.04 ± 1.22	***
CPD_adj_	4.51–5.82	5.22 ± 0.37	7.82–9.56	8.74 ± 0.49	***

Abbreviations: BD, body depth; CPD, caudal‐peduncle depth; HL, head length; OL, orbit length; PHL, postorbital head length; SNL, snout length; ST, standard length; TL, total length.

* *p* < .05;

** *p* < .01;

*** *p* < .001.

**FIGURE 3:**
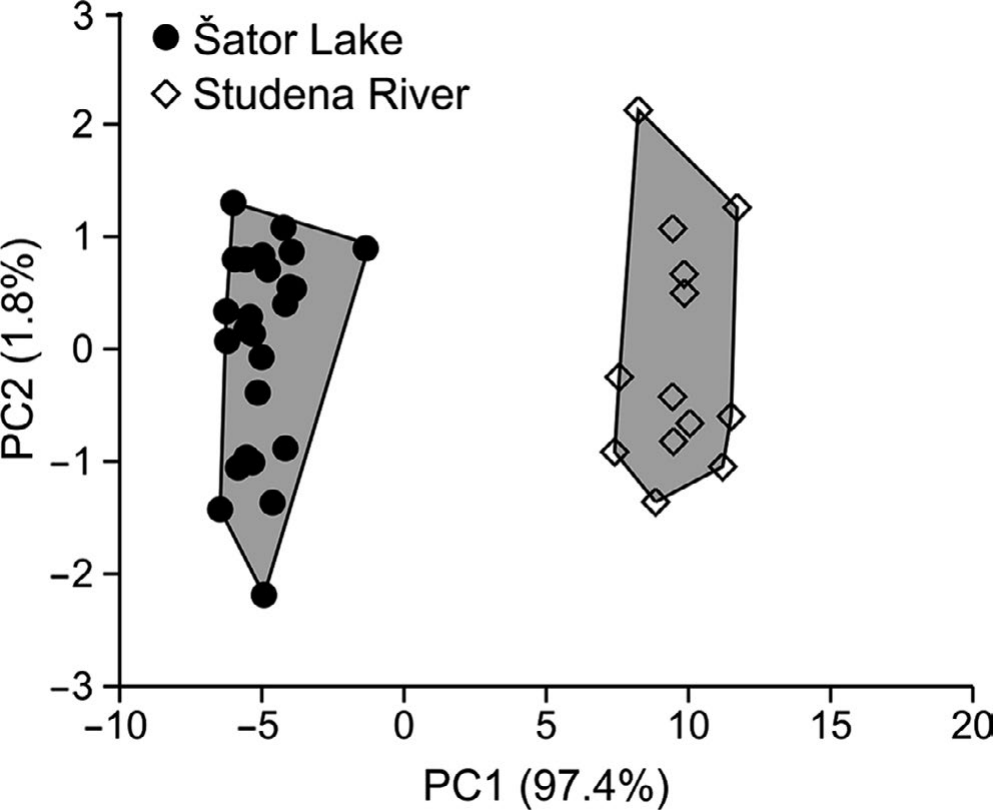
Plots of the first two principal component (PC) scores from PCA on size‐independent adjusted measurements of the *Aulopyge huegelii* populations. The percentage of explained variance of each PC is in parentheses

Nonparametric ANOVA performed on all types of linear measurements showed that two analyzed populations significantly differed in all but SNL(%HL) and PHL(%HL) (Table 1). PCA on size‐adjusted measures revealed that majority of the variation was explained with the first principal component (PC1 97.4%). Scatterplot for the first two PCs axes showed that those two populations clearly separated along PC1 (Figure 3). The measure with highest loading on PC1 was BD_adj_ (0.78).

### 3.2 Geometric morphometrics

Normality test showed that the size variable (centroid size) deviated from the normal distribution (Shapiro‐Wilk test, *p* < .05). Therefore, nonparametric ANOVA on centroid size was applied and showed no statistically significant differences between two populations (*F* = 0.026, *p* = .88). Multivariate regression of shape variables on centroid size showed no significant allometry (*p* = .34) and accounted for only 2.98% of the overall shape variation.

**FIGURE 4:**
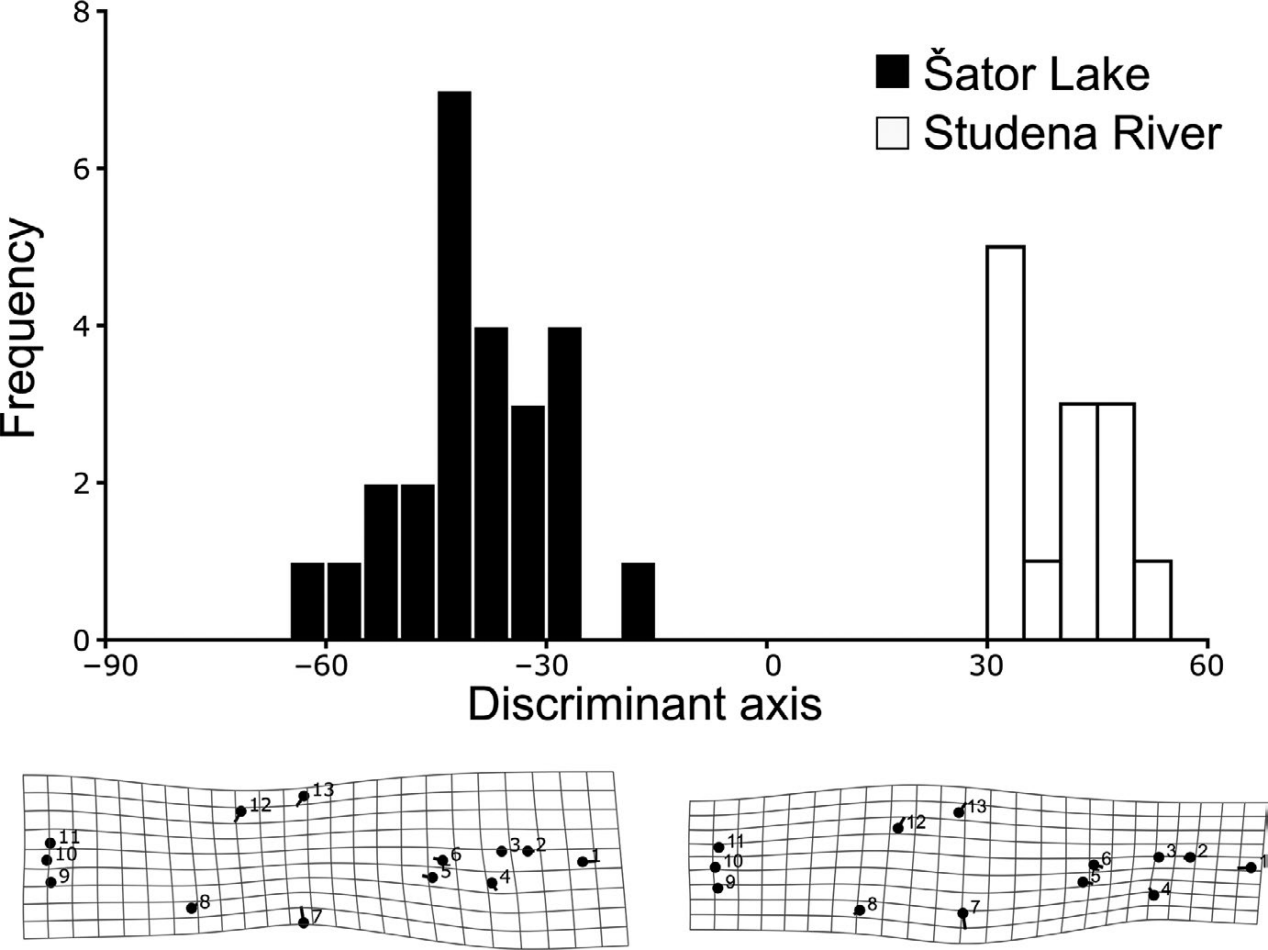
DFA histogram (above) and deformation grids of body shape differences between populations of *Aulopyge huegelii* (below). Numbers in the deformation grids refer to landmarks shown in Figure 1

Discriminant function analysis conducted on Procrustes coordinates showed significant body shape difference between populations (Procrustes distance = 0.0449, *p* < .0001) that were separated with no overlap along discriminant axis (Figure 4). Percentage of correct classification was 100% (92% after cross‐validation). Deformation grids indicated that displacement of landmarks 1, 7, 12, 13 had main influence on shape changes; individuals from Studena River are characterized by wider and slightly shorter body comparing to individuals from Šator Lake (Figure 4). Disparity analysis showed that Procrustes distance between the most divergent specimens within population was higher in Šator Lake (0.077) than in Studena River (0.066). Procrustes variances of two populations (Šator Lake: 0.00098; Studena River: 0.00106) did not differ significantly (*p* = .62).

### 3.3 Length‐weight relationship

The values of *r* ^2^ for both population were higher than 0.93. The obtained *b* value for Šator Lake is 2.490 (95% confidence limits: 1.987–2.994) and for Studena River is 2.922 (95% confidence limits: 2.443–3.401). The *t* test revealed statistically significant deviation from 3 for *b* value for population from Šator Lake (*p* < .05).

### 3.4 Molecular diversity

**FIGURE 5:**
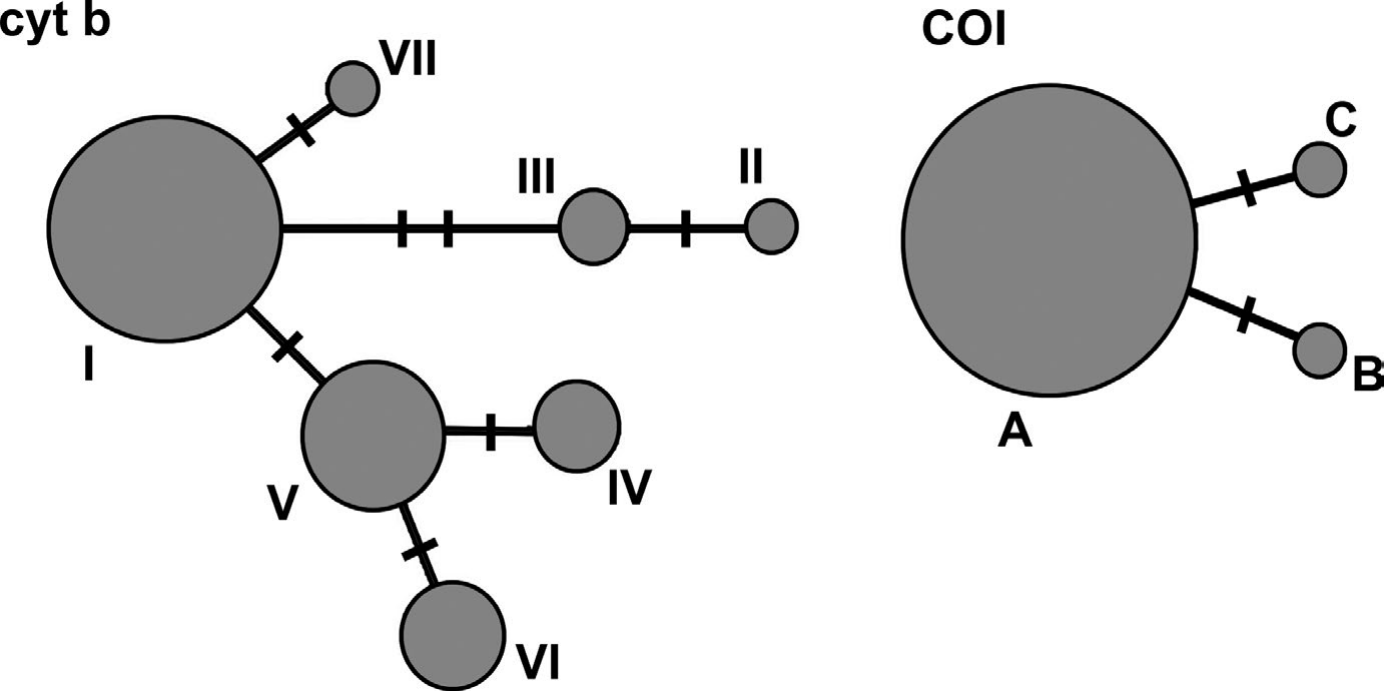
Spanning network of *cyt b* and *COI* mitochondrial DNA sequence haplotypes in *Aulopyge huegelii*. Each circle represents one haplotype, the size of the circle is proportional to the overall number of individuals with that haplotype. *Cyt b* mtDNA: HI—Šator Lake, Studena River; HII—Buško Lake; HIII—Šator Lake, Buško Lake; HIV—Šator Lake; HV: Šator Lake, Krka River; HVI and HVII: Studena River *COI* mtDNA: HA—Šator Lake, Studena River, Cetina river; HB—Cetina river; HC—Livno drainage. Thick marks on lines connecting haplotypes represent single‐nucleotide substitutions

A total of seven *cyt b* mtDNA haplotypes (around 1,110‐bp long) were found among the 39 analyzed individuals (sequences obtained in this study will be submitted to GenBank after the manuscript acceptance) (Figure 1; Table S2). There were seven variable positions defining *cyt b* mtDNA haplotypes (Table S3), diverging up to three bases from each other (Table S4). Four haplotypes (HI, HIII‐HV) were found in Šator Lake, three in Studena River (HI, HVI, HVII), whereas two and one were registered in Buško Lake (HII, HIII) and Krka River (HV), respectively. Unique haplotypes were found in Šator Lake (HIV), Studena River (HVI, HVII) and Buško Lake (HII) (Table S2; Figures 2 and 5). Intraspecific sequence divergences (uncorrected *p*‐distance as a percentage) ranged from 0.09% to 0.38% (Table S4).

Molecular variation of the 5′ *COI* mtDNA fragment (652‐bp long) of 37 fish encompassed three haplotypes (HA‐HC) with two variable positions in total, resulting in *p*‐distance 0.15%–0.31% (Table S5). All the 23 individuals from Šator Lake, ten from Studena River, and two from Cetina River shared the same haplotype (HA), while unique haplotypes B and C were registered in Cetina River and Livno drainage, respectively (Table S2; Figures 1 and 5).

The genetic heterogeneity and discordance in variation of the two mtDNA genes in Šator Lake and Studena River is in agreement with the *θ* values. Contrary to *cyt b* mtDNA that possess four (Šator Lake) and three (Studena River) distinct haplotypes, *COI* mtDNA showed a lack of variation in both populations. Hence, the highest *θ* values were observed for *cyt b* mtDNA in the Šator Lake sample (Table S6).

Using the Mantel's test no significant correlation between morphology (body shape) and genetics (*p*‐distance) was found (*R* = .04, *p* = .32).

## 4 DISCUSSION

In this study, we tested the usefulness of molecular markers (*COI* and *cyt b* mtDNA), and linear and geometric morphometrics in quantification of variation in populations with different evolutionary histories (natural – Studena River vs. introduced ‐ Šator Lake) and selection regimes (under‐ and aboveground ecosystems of sinking Studena River and strictly aboveground environment of Šator Lake) of *A. huegelii*, the relict cyprinid evolutionary lineage.

Contrary to *cyt b* mtDNA which possess four and three haplotypes in Šator Lake and Studena River, respectively, a lack of variation was observed at the barcoding fragment of *COI* mtDNA. Indeed, we revealed *cyt b* mtDNA as a faster evolving gene in this species, which is in concordance with the variation observed for *A. huegelii* from Buško Blato, Bosnia and Herzegovina (Mušović, 2016), and other endemic cyprinid species of *Telestes* (Buj et al., 2017; Gilles et al., 2010; Ketmaier et al., 2004; Perea et al., 2010), *Delminichthys* (Palandačić et al., 2012; Perea et al., 2010), and *Phoxinus* genera (Palandačić et al., 2015, 2017; Perea et al., 2010; Vucić et al., 2018) from the Dinaric karst. However, contrary to this study, *COI* mtDNA expressed variation in autochthonous populations of *Telestes metohiensis* and *T. dabar*, *Delminichthys* and *Phoxinus* genera (Francuski et al., 2019; Perea et al., 2010). Significant discordance in variation of two mtDNA genes once again advocates implementation of specific approaches and molecular markers for studying any evolutionary entity, which has already been proposed in the DNA barcoding debate (e.g., Krishnamurthy & Francis, 2012; Moritz & Cicero, 2004; Rubinoff, 2006; Sheth & Thaker, 2017).

Interpopulation differentiation of the two genes was observed in our study in spite of the limited DNA data that was available. Indeed, just a few *COI* mtDNA and *cyt b* mtDNA data were available for populations from the other localities on the Dinaric karst of Bosnia and Herzegovina (small rivers in Livanjsko Polje, Blidinje and Buško Lakes) and Croatia (Krka and Cetina Rivers) (Machordom & Doadrio, 2001; Tsigenopoulos & Berrebi, 2000). Interpopulation *cyt b* mtDNA variation observed in our study (Šator Lake vs. Studena River, *p*‐distance: 0.0%–0.28%) was in line with the differences obtained for other population comparisons (Buško Blato vs. Šator lake/Studena River/Krka River, *p*‐distance: 0.28%–0.38%; Krka River vs. Šator lake/Studena River/Buško Blato, *p*‐distance: 0.09%–0.38%). On the contrary, *COI* mtDNA as a slowly evolving gene expressed lower variation within *A. huegelii*. For instance, individuals from Šator Lake and Studena River shared the same haplotype (HA), which was also registered in Cetina River (Geiger et al., 2014), while HC found in Livno drainage (Geiger et al., 2014) slightly differed from HA (*p*‐distance: 0.15%) and HB (*p*‐distance: 0.31%).

Contrasting variation of the two molecular markers was shown by *θ* values as well. The genetic heterogeneity at *cyt b* mtDNA expressed by *θ_K_*, *θ_H_* and *θ_S_* was slightly higher in Šator Lake compared to Studena River. However, introduced population possessed more *cyt b* haplotypes (I, III, IV, V) than natural populations (I, VI, VII), which is in contrast to the number of private haplotypes (Šator lake: IV; Studena River: VI, VII). Since the Šator lake population shares the same haplotypes with Studena River (I), Buško Blato (III), and Krka River (V) we assumed that more than once Dalmatian barbelgudgeon has been introduced to the novel environment. Indeed, data on origin and number of introduced fish is still unknown (Delić et al., 2005), further step of spatial analyses of molecular and phenotypic variation that will include more individuals and samples would provide better understanding of this subject.

Furthermore, by testing linear and landmark‐based geometric morphometric approaches in quantifying phenotypic variation between the two populations of *A. huegelli* we found contrasting results. Phenotypic divergence was found to be statistically significant considering both raw and transformed (allometry‐free) linear measurements. So far, linear measurements were used in study of phenotypic variation of the Dalmatian barbelgudgeon from Buško Blato (Guzina, 2000; Mušović et al., 2018) and Ždralovac canal in Bosnia and Herzegovina, and Čikola River, Visovac and Torak Lakes in Croatia (Mihinjač, 2018), but spatial pattern across the entire species area remains uncover. Comparing measurements used in these studies (see Mihinjač, 2018; Mušović et al., 2018) and sample from Studena River (Duvanjsko Polje), we found similarity in mean values as well as that ranges (min‐max values) were greatly overlapped for all measurements we reported. Contrary to already analyzed natural (autochthonous) populations (Mihinjač, 2018; Mušović et al., 2018) and Studena River (data herewith), some individuals of the introduced sample attain smaller body size influencing smaller mean and minimal values of the studied traits of the Šator Lake sample. The larger body dimensions of fish in the river habitat compared to those in the lake we found in this study contradict the assumption that in lake habitats, due to uniform environmental conditions throughout the year and available food, larger individuals develop (Mihinjač, 2018). Indeed, Mihinjač (2018) reported higher body length for *A. huegelli* from lake systems than from Čikola River. However, standardized linear measurements independent to SL obtained through allometric transformation revealed that fish from Čikola River are characterized by longer head length in relation to lake individuals (Mihinjač, 2018) which is consistent with our findings. In addition, length‐weight relationship was calculated for the *A. huegelii* populations from Croatia and the obtained *b* parameter values (3.322, Treer et al., 2008; 3.0166 and 3.1354 for Čikola River and lake systems, respectively, Mihinjač, 2018) were higher than our estimates (2.922 and 2.490 for Šator Lake and Studena River, respectively). However, 95% confidence limits of *b* for *A. huegelii* from Čikola River and lake systems was ranged from 2.9442 to 3.0890 and 2.8802 to 3.3905, respectively, indicating that it includes the isometric value 3 (Mihinjač, 2018), as we also found for Studena River (2.443–3.401) but not for Šator Lake where upper limit of 95% confidence interval was close to 3 (1.987–2.994). Lower *b* values we found and deviation from isometric growth for population from Šator Lake are possibly due to small sample size and standard length range covered by sample (Froese, 2006). Also, variability of the parameter *b* in some species is in relation with different sampling seasons and spawning period (Treer et al., 2008). It should take into account that this lack of consistency with published data regarding the difference in body size between habitats could be the result of year‐on‐year variation of specific environmental conditions and available food. For example, comparing morphometric data on *A. huegelii* populations from Buško Blato collected in period 1966–2015 revealed significant differences between temporal samples in body length and mass (Guzina, 2000; Mušović et al., 2018). Since being collected in different years (Šator Lake in 2015, Studena River in 2017), relationship in body size of lake and river population we found does not necessarily reflect the deviation from general pattern, but rather can be an indicator of ecological habitat conditions at the time of sampling.

Contrary to almost all studied linear measurements, no significant difference in centroid size obtained by geometric morphometric approach was observed. However, phenotypic dissimilarity was found regarding body shape, suggesting that changes were mainly associated with displacement of landmarks which influenced the body depth and head length in individuals. As far as we know, this is first study which implemented geometric morphometrics in understanding pattern of phenotypic variation of the Dalmatian barbelgudgeon. However, geometric morphometrics has been approved as a valuable tool for uncovering subtle body shape divergence among conspecific populations of European freshwater fish underlying the influence of heterogeneous ecological environments (e.g., Bajić et al., 2018; Collin & Fumagalli, 2011, 2015; Francuski et al., 2019; Marić et al., 2015; Ramler et al., 2017; Zaccara et al., 2019; Závorka et al., 2020). Namely, it was found that differences in body shape associated with environmental factors result in a morphologically optimized phenotype for a given habitat such as a more streamline (slender) body shape and larger head of lake fish compare to stream ones (Ramler et al., 2017) as we also found. The observed shape differences are likely closely linked to swimming and feeding performances, and predatory pressure in different habitats (Ramler et al., 2017). In addition, based on a very few available ecological data (physico‐chemical parameters) for the Šator Lake and Studena River (Table S1) difference between these two sites, primarily in water temperature and dissolved oxygen concentration, is noticed. It has already known that oxygen availability, temperature as well as elevation could influence morphological changes of cyprinids (Collin & Fumagalli, 2011). Therefore, it is (again) important to highlight that recognizing morphological differentiation of the adaptive traits, such as shape divergence obtained in this study, mirrors effect of divergent local natural selection on populations from heterogeneous habitats. Considering recent introduction of Dalmatian barbelgudgeon to Šator Lake, which flows into the Unac River, and thus, belongs to the Una River and the Black Sea basin, this population has specific evolutionary history, biogeography, and habitats. Indeed, during over 40 years since its introduction, *A. huegelii* has adapted to the extreme environment (Šator Lake is situated at the highest altitude within the distribution area of the species, 1,488 m a.s.l.), unlike the introduced trout species that did not manage to survive (Delić et al., 2005).

Finally, given that the uniqueness of genetic, morphological, ecological, and life‐history traits of *A. huegelli* provide valuable information on its distinct evolutionary history, broader study of the species across the distribution area is essential for setting conservation priorities and understanding the evolutionary history and phylogenetic diversity of the family Cyprinidae. This is of high relevance since assessment of taxonomic diversity is a prerequisite for indentifying priority areas of the conservation interest such as the Balkan karst region, which according to Reed et al. (2004) represents one of the most famous karstic regions of the world. As such, it is highlighted as the hotspot of freshwater fish biodiversity.

## CONFLICT OF INTEREST

The authors declare no conflict of interest.

## AUTHOR CONTRIBUTION

**Jasmina Ludoški:** Data curation (equal); Formal analysis (equal); Investigation (equal); Methodology (equal); Visualization (equal); Writing‐original draft (equal); Writing‐review & editing (equal). **Ljubinka Francuski:** Conceptualization (equal); Data curation (equal); Formal analysis (equal); Investigation (equal); Methodology (equal); Visualization (equal); Writing‐original draft (equal); Writing‐review & editing (equal). **Milica Lukač:** Conceptualization (equal); Data curation (equal); Formal analysis (equal); Investigation (equal); Visualization (equal); Writing‐original draft (equal); Writing‐review & editing (equal). **Radoslav Dekić:** Data curation (equal); Writing‐review & editing (equal). **Vesna Milankov:** Conceptualization (equal); Funding acquisition (equal); Investigation (equal); Methodology (supporting); Project administration (lead); Resources (equal); Supervision (lead); Writing‐original draft (equal); Writing‐review & editing (equal).

## Supporting information

Supplementary Material

## Data Availability

The DNA sequences analyzed in the manuscript have been archived in GenBank, while the other data supporting the results and conclusions were included in the additional files of the article. GenBank accessions: *cyt b* mtDNA: MT921920‐MT921955; *COI* mtDNA: MT920042‐MT920074.

## References

[ece37108-bib-0001] Armbruster, J. W. (2012). Standardized measurements, landmarks, and meristic counts for cypriniform fishes. Zootaxa, 3586, 8–16. 10.11646/zootaxa.3586.1.3

[ece37108-bib-0002] Bajić, A. , Jojić, V. , Snoj, A. , Miljanović, B. , Askeyev, O. , Askeyev, I. , & Marić, S. (2018). Comparative body shape variation of the European grayling *Thymallus thymallus* (Actinopterygii, Salmonidae) from wild populations and hatcheries. Zoologischer Anzeiger, 272, 73–80. 10.1016/j.jcz.2017.12.005

[ece37108-bib-0003] Benovics, M. , Kičinjaová, M. L. , & Šimková, A. (2017). The phylogenetic position of the enigmatic Balkan *Aulopyge huegelii* (Teleostei: Cyprinidae) from the perspective of host‐ specific *Dactylogyrus* parasites (Monogenea), with a description of *Dactylogyrus omenti* n. sp. Parasites and Vectors, 10, 547 10.1186/s13071-017-2491-z 29100541 PMC5670733

[ece37108-bib-0004] Bless, R. , & Riehl, R. (2002). Biology and egg morphology of the Dalmatian barbelgudgeon *Aulopyge huegeli*, an endangered endemic species in Croatia. Environmental Biology of Fishes, 63(4), 451–456.

[ece37108-bib-0005] Buj, I. , Marčić, Z. , Ćaleta, M. , Šanda, R. , Geiger, M. F. , Freyhof, J. , Machordom, A. , & Vukić, J. (2017). Ancient connections among the European rivers and watersheds revealed from the evolutionary history of the genus *Telestes* (Actinopterygii; Cypriniformes). PLoS One, 12(12), e0187366 10.1371/journal.pone.0187366 29227999 PMC5724836

[ece37108-bib-0006] Ćaleta, M. , Buj, I. , Mrakovčić, M. , Mustafić, P. , Zanella, D. , Marčić, Z. , Duplić, A. , Mihinjač, T. , & Katavić, I. (2015). Hrvatske endemske ribe (116 str.). Agencija za zaštitu okoliša. (In Serbian).

[ece37108-bib-0007] Ćaleta, M. , Marčić, Z. , Buj, I. , Zanella, D. , Mustafić, P. , Duplić, A. , & Horvatić, S. (2019). A review of extant Croatian freshwater fish and lampreys ‐ Annotated list and distribution. Croatian Journal of Fisheries, 77, 137–234. 10.2478/cjf-2019-0016

[ece37108-bib-0008] Ćaleta, M. , Mrakovčić, M. , Buj, I. , Mustafić, P. , Zanella, D. , & Marčić, Z. (2009). Threatened fishes of the world: *Aulopyge huegelii* Heckel, 1842 (Cyprinidae). Environmental Biology of Fishes, 85(1), 21–22. 10.1007/s10641-009-9445-z

[ece37108-bib-0009] Collares‐Pereira, M. J. (1994). The karyology of barbins and the plesiomorphic condition of polyploidy in Cyprinidae. Bulletin Français de la Peche et de la Pisciculture, 334, 191–199.

[ece37108-bib-0010] Collin, H. , & Fumagalli, L. (2011). Evidence for morphological and adaptive genetic divergence between lake and stream habitats in European minnows (*Phoxinus phoxinus*, Cyprinidae). Molecular Ecology, 20(21), 4490–4502. 10.1111/j.1365-294X.2011.05284.x 21951706

[ece37108-bib-0011] Collin, H. , & Fumagalli, L. (2015). The role of geography and ecology in shaping repeated patterns of morphological and genetic differentiation between European minnows (*Phoxinus phoxinus*) from the Pyrenees and the Alps. Biological Journal of the Linnean Society, 116(3), 691–703.

[ece37108-bib-0012] Crivelli, A. J. (2006). Aulopyge huegelii The IUCN Red List of Threatened Species 2006: e.T61350A12466288.

[ece37108-bib-0013] Dekić, R. , Bilbija, B. , Lukač, M. , Mandić, M. , Friščić, J. , Ivanc, A. , & Bećiraj, A. (2016). Morfometrijske karakteristike oštrulja (*Aulopyge huegelii*) i pijurice (*Phoxinellus alepidotus)* iz Šatorskog jezera. Skup, 7(2), 123–128.

[ece37108-bib-0014] Delić, A. , Kučinić, M. , Marić, D. , & Bučar, M. (2005). New data about the distribution of *Phoxinellus alepidotus* (Heckel, 1843) and *Aulopyge huegelii* (Heckel, 1841). Natura Croatica: Periodicum Musei Historiae Naturalis Croatici, 14(4), 351–355.

[ece37108-bib-0015] Dryden, I. L. , & Mardia, K. V. (1998). Statistical shape analysis. Wiley.

[ece37108-bib-0016] Elliot, N. G. , Haskard, K. , & Koslow, J. A. (1995). Morphometric analysis of orange roughy (*Hoplostethus atlanticus*) off the continental slope of southern Australia. Journal of Fish Biology, 46, 202–220. 10.1111/j.1095-8649.1995.tb05962.x

[ece37108-bib-0017] Excoffier, L. , Laval, G. , & Schneider, S. (2005). Arlequin (version 3.0): An integrated software package for population genetics data analysis. Evolutionary Bioinformatics, 1, 47–50. 10.1177/117693430500100003 PMC2658868 19325852

[ece37108-bib-0018] Folmer, O. , Black, M. , Hoeh, W. , Lutz, R. , & Vrijenhoek, R. (1994). DNA primers for amplification of mitochondrial cytochrome *c* oxidase subunit I from diverse metazoan invertebrates. Molecular Marine Biology and Biotechnology, 3, 294–299. 7881515

[ece37108-bib-0019] Francuski, L. , Ludoški, J. , Lukač, M. , Dekić, R. , & Milankov, V. (2019). Integrative study of population structure of *Telestes dabar*, the strictly endemic cyprinid species from the Dinaric karst on the Balkan Peninsula. European Journal of Wildlife Research, 65, 66 10.1007/s10344-019-1302-6

[ece37108-bib-0020] Froese, R. (2006). Cube law, condition factor and weight‐length relationships: History, meta‐analysis and recommendations. Journal of Applied Ichthyology, 22, 241–253. 10.1111/j.1439-0426.2006.00805.x

[ece37108-bib-0021] Geiger, M. F. , Herder, F. , Monaghan, M. T. , Almada, V. , Barbieri, R. , Bariche, M. , Berrebi, P. , Bohlen, J. , Casal‐Lopez, M. , Delmastro, G. B. , Denys, G. P. J. , Dettai, A. , Doadrio, I. , Kalogianni, E. , Kärst, H. , Kottelat, M. , Kovačić, M. , Laporte, M. , Lorenzoni, M. , … Freyhof, J. (2014). Spatial heterogeneity in the Mediterranean Biodiversity Hotspot affects barcoding accuracy of its freshwater fishes. Molecular Ecology Resources, 14(6), 1210–2122. 10.1111/1755-0998.12257 24690331

[ece37108-bib-0022] Gilles, A. , Costedoat, C. , Barascud, B. , Voisin, A. , Banarescu, P. , Bianco, P. G. , & Chappaz, R. (2010). Speciation pattern of *Telestes souffia* complex (Teleostei, Cyprinidae) in Europe using morphological and molecular markers. Zoologica Scripta, 39, 225–242.

[ece37108-bib-0023] Guzina, N. (2000). Morfološko –taksonomske karakteristike vrste *Aulopyge hügeli* Hekel, 1841. PhD thesis, University of Sarajevo, Bosnia and Hercegovina.

[ece37108-bib-0024] Hall, T. A. (1999). BioEdit: A user‐friendly biological sequence alignment editor and analysis program for Windows 95/98/NT. Nucleic Acid Symposium Series, 41, 95–98.

[ece37108-bib-0025] Hammer, Ø. , Harper, D. A. T. , & Ryan, P. D. (2001). PAST: Paleontological statistics software package for education and data analysis. Palaeontologia Electronica, 4(1), 1–9.

[ece37108-bib-0026] Howes, G. J. (1987). The phylogenetic position of the Yugoslavian cyprinid fish genus *Aulopyge* Heckel, 1841, with an appraisal of the genus *Barbus* Cuvier and Cloquet, 1816, and the subfamily Cyprininae. Bulletin of the British Museum (Natural History), 52, 165–196.

[ece37108-bib-0027] Isaac, N. J. B. , Turvey, S. T. , Collen, B. , Waterman, C. , & Baillie, J. E. M. (2007). Mammals on the EDGE: Conservation priorities based on threat and phylogeny. PLoS One, 2(3), e296 10.1371/journal.pone.0000296 17375184 PMC1808424

[ece37108-bib-0028] Ketmaier, V. , Bianco, P. G. , Coboli, M. , Krivokapic, M. , Caniglia, R. , & De Matthaeis, E. (2004). Molecular phylogeny of two lineages of Leuciscinae cyprinids (*Telestes* and *Scardinius*) from peri‐Mediterranean area based on cytochrome *b* data. Molecular Phylogenetics and Evolution, 32, 1061–1071. 15288075 10.1016/j.ympev.2004.04.008

[ece37108-bib-0029] Klingenberg, C. P. (2011). MorphoJ: An integrated software package for geometric morphometrics. Molecular Ecology Resources, 11, 353–357. 10.1111/j.1755-0998.2010.02924.x 21429143

[ece37108-bib-0030] Klingenberg, C. P. , & McIntyre, G. S. (1998). Geometric morphometrics of developmental instability: Analysing patterns of fluctuating asymmetry with Procrustes methods. Evolution, 52(5), 1363–1375. 28565401 10.1111/j.1558-5646.1998.tb02018.x

[ece37108-bib-0031] Kottelat, M. , & Freyhof, J. (2007). Telestes. Handbook of European freshwater fishes. Kottelat.

[ece37108-bib-0032] Krishnamurthy, P. K. , & Francis, R. A. (2012). A critical review on the utility of DNA barcoding in biodiversity conservation. Biodiversity Conservation, 21, 1901–1919. 10.1007/s10531-012-0306-2

[ece37108-bib-0033] Kumar, S. , Stecher, G. , Li, M. , Knyaz, C. , & Tamura, K. (2018). MEGA X: Molecular evolutionary genetics analysis across computing platforms. Molecular Biology and Evolution, 35, 1547–1549. 10.1093/molbev/msy096 29722887 PMC5967553

[ece37108-bib-0034] Le Cren, E. D. (1951). The length‐weight relationship and seasonal cycle in gonad weight and condition in the perch (*Perca fluviatilis*). Journal of Animal Ecology, 20, 201–219. 10.2307/1540

[ece37108-bib-0035] Machordom, A. , & Doadrio, I. (2001). Evolutionary history and speciation modes in the cyprinid genus *Barbus* . Proceedings of the Royal Society B, 268, 1297–1306. 11410158 10.1098/rspb.2001.1654 PMC1088741

[ece37108-bib-0037] Marić, S. , Snoj, A. , Krpo‐Ćetković, J. , Šanda, R. , & Jojić, V. (2015). Genetic and morphological variability of the European mudminnow *Umbra krameri* (Teleostei, Umbridae) in Serbia and in Bosnia and Herzegovina, a basis for future conservation activities. Journal of Fish Biology, 86, 1534–1548. 25801689 10.1111/jfb.12657

[ece37108-bib-0038] Mihinjač, T. (2018). Bio – ecological characteristics of Dalmatian barbelgudgeon *Aulopyge huegelii* Heckel, 1843 (Cyprinidae; Actinopterygii). PhD thesis, University of Zagreb, Croatia. (In Croatian).

[ece37108-bib-0039] Milankov, V. , Ludoški, J. , Ståhls, G. , Stamenković, J. , & Vujić, A. (2009). High molecular and phenotypic diversity in the *Merodon avidus* complex (Diptera, Syrphidae): Cryptic speciation in a diverse insect taxon. Zoological Journal of the Linnean Society, 155, 819–833.

[ece37108-bib-0040] Moritz, C. , & Cicero, C. (2004). DNA barcoding: Promis and pitfalls. PloS Biology, 2, 1529–1531. 10.1371/journal.pbio.0020354 PMC519004 15486587

[ece37108-bib-0041] Mrakovčić, M. , & Mišetić, S. (1990). Status, distribution and conservation of the salmonid, *Salmothymus obtusirostris* (Heckel) and the cyprinid, *Aulopyge hugelii* (Heckel) in Yugoslavia. Journal of Fish Biology, 37, 241–242.

[ece37108-bib-0042] Mušović, A. (2016). Ekološka, morfološka i molekularno‐genetička karakterizacija vrste *Aulopyge huegelii* Heckel, 1843 (Actinopterygii: Cipriniformes) iz Buškog jezera. PhD thesis, University of Sarajevo, Bosnia and Hercegovina.

[ece37108-bib-0043] Mušović, A. , Đug, S. , Pojskić, N. , Kalamujić Stroil, B. , Vesnić, A. , & Škrijelj, R. (2018). Status of endangered fish species *Aulopyge huegelii* Heckel, 1843 (Teleostei: Cyprinidae) in the Buško Blato reservoir, Bosnia and Herzegovina. Iranian Journal of Ichthyology, 5(3), 212–231.

[ece37108-bib-0044] Oikonomou, A. , Leprieur, F. , & Leonardos, I. D. (2014). Biogeography of freshwater fishes of the Balkan Peninsula. Hydrobiologia, 2014(738), 205–220.

[ece37108-bib-0045] Palandačić, A. , Bravničar, J. , Zupančič, P. , Šanda, R. , & Snoj, A. (2015). Molecular data suggest a multispecies complex of *Phoxinus* (Cyprinidae) in the western Balkan Peninsula. Molecular Phylogenetics and Evolution, 92, 118–123. 10.1016/j.ympev.2015.05.024 26143109

[ece37108-bib-0046] Palandačić, A. , Matschiner, M. , Zupančič, P. , & Snoj, A. (2012). Fish migrate underground: The example of *Delminichthys adspersus* (Cyprinidae). Molecular Ecology, 21(7), 1658–1671. 22369427 10.1111/j.1365-294X.2012.05507.x

[ece37108-bib-0047] Palandačić, A. , Naseka, A. , Ramler, D. , & Ahnelt, H. (2017). Contrasting morphology with molecular data: An approach to revision of species complexes based on the example of European *Phoxinus* (Cyprinidae). BMC Evolutionary Biology, 17, 184 10.1186/s12862-017-1032-x 28793855 PMC5549366

[ece37108-bib-0048] Perea, S. , Böhme, M. , Zupančič, P. , Freyhof, J. , Šanda, R. , Özuluğ, M. , Abdoli, A. , & Doadrio, I. (2010). Phylogenetic relationships and biogeographical patterns in Circum‐Mediterranean subfamily Leuciscinae (Teleostei, Cyprinidae) inferred from both mitochondrial and nuclear data. BMC Evolutionary Biology, 10, 265 10.1186/1471-2148-10-265 20807419 PMC2940817

[ece37108-bib-0049] Radoš, D. , Lozić, S. , & Siljeg, A. (2012). Morphometrical characteristics of the broader area of Duvanjsko Polje, Bosnia and Herzegovina. Geoadria, 17(2), 177–207.

[ece37108-bib-0050] Ramler, D. , Palandačić, A. , Delmastro, G. B. , Wanzenböck, J. , & Ahnelt, H. (2017). Morphological divergence of lake and stream *Phoxinus* of Northern Italy and the Danube basin based on geometric morphometric analysis. Ecology and Evolution, 7(2), 572–584. 28116054 10.1002/ece3.2648 PMC5243779

[ece37108-bib-0051] Reed, J. M. , Krystufek, B. , & Eastwood, W. J. (2004). The physical geography of the Balkans and nomenclature of place names In Griffiths H. I., Kryštufek B., & Reed J. M. (Eds.), Balkan biodiversity (pp. 9–22). Kluwer Academic Publishers.

[ece37108-bib-0052] Ritter‐Studnićka, H. , & Grgić, P. (1971). Die Reste der Stieleichenwälder in Livanjsko Pol‐je(Bosnien). Botanische Jahrbücher für Systematik, Pflanzengeschichte und Pflan‐zengeographie, 91(2/3), 330–347.

[ece37108-bib-0053] Rohlf, F. J. (2017). TpsDig2, version 2.31. Department of Ecology and Evolution, State University of New York at Stony Brook.

[ece37108-bib-0054] Rubinoff, D. (2006). Utility of mitochondrial DNA barcodes in species conservation. Conservation Biology, 20(4), 1026–1033. 16922219 10.1111/j.1523-1739.2006.00372.x

[ece37108-bib-0055] Sheets, H. D. (2007). Disparity Box, IMP. Canisius College, Buffalo, NY.

[ece37108-bib-0056] Sheth, B. P. , & Thaker, V. S. (2017). DNA barcoding and traditional taxonomy: An integrated approach for biodiversity conservation. Genome, 60(7), 618–628. 10.1139/gen-2015-0167 28431212

[ece37108-bib-0057] Treer, T. , Šprem, N. , Torcu‐Koc, H. , Sun, Y. , & Piria, M. (2008). Length‐weight relationships of freshwater fishes of Croatia. Journal of Applied Ichthyology, 24, 626–628. 10.1111/j.1439-0426.2008.01084.x

[ece37108-bib-0058] Tsigenopoulos, C. S. , & Berrebi, P. (2000). Molecular phylogeny of North Mediterranean freshwater fauna (genus *Barbus*: Cyprinidae) inferred from cytochrome *b* sequences: Biogeographic and systematic implications. Molecular Phylogenetics and Evolution, 14, 165–179. 10679153 10.1006/mpev.1999.0702

[ece37108-bib-0059] Vucić, M. , Jelić, D. , Žutinić, P. , Grandjean, F. , & Jelić, M. (2018). Distribution of Eurasian minnows (*Phoxinus*: Cypriniformes) in the Western Balkans. Knowledge and Management of Aquatic Ecosystems, 419, 11.

[ece37108-bib-0060] Vuković, T. , & Ivanović, B. (1971). Freshwater fishes of Yugoslavia. Zemaljski Muzej.

[ece37108-bib-0061] Wang, J. , Wu, X. , Chen, Z. , Yue, Z. , Ma, W. , & Luo, J. (2013). Molecular phylogeny of European and African *Barbus* and their west Asian relatives in the Cyprininae (Teleostei: Cyprinoformes) and orogenesis of the Quinghai‐ Tibetian plateau. Chinese Science Bulletin, 58, 3738–3746.

[ece37108-bib-0063] Zaccara, S. , Quadroni, S. , De Santic, V. , Vanetti, I. , Carosi, A. , Britton, R. , & Lorezoni, M. (2019). Genetic and morphological analyses reveal a complex biogeographic pattern in the endemic barbel populations of the southern Italian peninsula. Ecology & Evolution, 9(18), 10185–10197. 10.1002/ece3.5521 31624544 PMC6787835

[ece37108-bib-0065] Zardoya, R. , & Doadrio, I. (1998). Phylogenetic relationships of Iberian cyprinids: Systematic and biogeographical implications. Proceedings of the Royal Society B: Biological Sciences, 265(1403), 1365–1372. 10.1098/rspb.1998.0443 PMC1689212 9718739

[ece37108-bib-0066] Závorka, L. , Larranaga, N. , Lovén Wallerius, M. , Näslund, J. , Koeck, B. , Wengström, N. , Cucherousset, J. , & Johnsson, J. I. (2020). Within‐stream phenotype divergence in head shape of brown trout associated with invasive brook trout. Biological Journal of the Linnean Society, 129(2), 347–355.

[ece37108-bib-0067] Zelditch, M. L. , Swiderski, D. L. , & Sheets, H. D. (2012). Geometric morphometrics for biologists: A primer (2nd ed.). Elsevier Academic Press.

