# Supplementary material for: Toward the conservation of the endemic monotypic fish genus Aulopyge from the Balkan Dinaric karst: Integrative assessment of introduced and natural population
Source: Ecol Evol. 2020 Dec 10;11(2):688–99. doi: 10.1002/ece3.7108 (PMC7820161; doi:10.1002/ece3.7108)
Supplement: Supplementary file 1 — Supplementary Material [file ECE3-11-688-s001.doc]

**Toward the conservation of the endemic monotypic fish genus *Aulopyge* from the Balkan Dinaric karst: Integrative assessment of introduced and natural population**

Jasmina Ludoški^1^, Ljubinka Francuski^1,2^, Milica Lukač^3^, Radoslav Dekić^3^, Vesna Milankov^1*^

^1^Faculty of Sciences, Department of Biology and Ecology, University of Novi Sad, Novi Sad, Serbia

^2^Present address: Groningen Institute for Evolutionary Life Sciences, University of Groningen, Groningen, The Netherlands

# ^3^Faculty of Sciences, University of Banja Luka, Banja Luka, Republic of Srpska, Bosnia and Herzegovina

Jasmina Ludoški and Ljubinka Francuski contributed equally to this work.

Correspondence:

Vesna Milankov

Faculty of Sciences, Department of Biology and Ecology, University of Novi Sad, Trg Dositeja Obradovića 2, 21000 Novi Sad, Serbia

Email: vesna.milankov@dbe.uns.ac.rs

| Table S1. Water quality parameters available for Studena River and Šator Lake (* Drina River is a small temporary flowing watercourse in Duvanjsko Field formed by joining of Studena River and Miljacka River; na- data not available). | | | | | | | | |
| --- | --- | --- | --- | --- | --- | --- | --- | --- |
|  | Studena River September 2018  (Dr Jerko Pavličević, personal communication) | Drina River* March 2019  (Agency for watershed of the Adriatic sea, Mostar, Bosnia and Herzegovina) | Drina River Decembre 2019  (Agency for watershed of the Adriatic sea, Mostar, Bosnia and Herzegovina) | Šator Lake  August 2015  (collected by M. Lukač and R. Dekić) | | | | |
|  |  |  |  | Point 1 | Point 2 | Point 3 | Point 4 | Point 5 |
| Water temperature (°C) | 14.7 | 11.7 | 10.2 | 16.3 | 16.0 | 8.1 | 17.5 | 16.8 |
| pH | 8.49 | 8.2 | 7.8 | 9.48 | 9.57 | 7.55 | 9.21 | 9.42 |
| Conductivity (uS/cm) | 318 | 308 | 319 | 155.5 | 209 | 318 | 159.6 | 212 |
| Total Dissolved Solids (mg/L) | 319 | na | na | na | na | na | na | na |
| Total Suspended Solids (mg/L) | na | 0.90 | <0.1 | na | na | na | na | na |
| Turbidity (NTU) | na | na | na | 0.91 | 0.75 | 0.83 | 1.04 | 0.80 |
| Dissolved oxygen (mg/l) | 10.3 | 12.05 | 12.42 | 8.43 | 9.64 | 9.91 | 8.41 | 8.88 |
| Oxygen saturation (%) | 99 | 111.1 | 110.5 | 102.6 | 116.7 | 99.9 | 105.2 | 109.5 |
| BOD_5_ (mg/L) | na | 0.61 | 0.94 | na | na | na | na | na |
| Permanganate Index (mg O2/L) | 3.72 | 0.89 | 1.14 | na | na | na | na | na |
| NH4-N (mg/L) | 0.151 | <0.039 | <0.039 | na | na | na | na | na |
| NO3-N (mg/L) | 0.31 | <0.220 | <0.220 | na | na | na | na | na |
| NO2-N (mg/L) | <0.01 | na | na | na | na | na | na | na |
| Total-N (mg/L) | 1.76 | 0.13 | 0.127 | na | na | na | na | na |
| Total-P (mg/L) | 0.026 | 0.007 | 0.020 | na | na | na | na | na |
| Orto-P (mg/L) | na | <0.005 | 0.008 | na | na | na | na | na |
| Chloride (mg/L) | na | 2.1 | 3.5 | na | na | na | na | na |
| Sulfate (mg/L) | na | 3.69 | 2.36 | na | na | na | na | na |
| TOC (mg/L) | na | 1.46 | 0.29 | na | na | na | na | na |
| Copper (μg/L) | na | 4.49 | 2.35 | na | na | na | na | na |
| Chromium (μg/L) | na | 1.74 | 0.30 | na | na | na | na | na |
| Zinc (μg/L) | na | 30.40 | 74.90 | na | na | na | na | na |
| Total coliform bacteria (no/100) | na | 2400 | 47 | na | na | na | na | na |

| Table S2. Details of sequence data of *Aulopyge huegelii* used in this study. | | | | | | |
| --- | --- | --- | --- | --- | --- | --- |
| Locality | Source study | ID | *cyt b* mtDNA | | *COI* mtDNA | |
|  |  |  | GenBank Acc. number | Haplotype | GenBank Acc. number | Haplotype |
| Šator Lake (Livansko Polje),  Bosnia and Herzegovina | This study | R76 | MT921920 | I | MT920042 | A |
|  |  | R77 | MT921921 | I | MT920043 | A |
|  |  | R78 | MT921922 | IV | MT920044 | A |
|  |  | R79 | MT921923 | I | MT920045 | A |
|  |  | R80 | MT921922 | I | MT920046 | A |
|  |  | R81 | MT921925 | I | MT920047 | A |
|  |  | R82 | MT921926 | I | MT920048 | A |
|  |  | R83 | MT921927 | I | MT920049 | A |
|  |  | R84 | MT921928 | IV | MT920050 | A |
|  |  | R85 | MT921929 | I | MT920051 | A |
|  |  | R86 | MT921930 | V | MT920052 | A |
|  |  | R87 | MT921931 | III | - | - |
|  |  | R88 | MT921932 | IV | MT920053 | A |
|  |  | R89 | MT921933 | I | MT920054 | A |
|  |  | R90 | MT921934 | I | MT920055 | A |
|  |  | R91 | MT921935 | V | MT920056 | A |
|  |  | R92 | MT921936 | V | MT920057 | A |
|  |  | R93 | MT921937 | V | MT920058 | A |
|  |  | R94 | MT921938 | I | MT920059 | A |
|  |  | R95 | MT921939 | V | MT920060 | A |
|  |  | R96 | MT921940 | I | MT920061 | A |
|  |  | R97 | MT921941 | V | MT920062 | A |
|  |  | R98 | MT921942 | I | MT920063 | A |
|  |  | R99 | MT921943 | V | MT920064 | A |
| Studena River (Duvanjsko Polje),  Bosnia and Herzegovina | This study | R139 | MT921944 | VI | MT920065 | A |
|  |  | R140 | MT921945 | VI | - | - |
|  |  | R141 | MT921946 | VI | - | - |
|  |  | R142 | MT921947 | I | MT920066 | A |
|  |  | R143 | - | - | MT920067 | A |
|  |  | R144 | MT921948 | I | MT920068 | A |
|  |  | R145 | MT921949 | I | MT920069 | A |
|  |  | R146 | MT921950 | I | - | - |
|  |  | R147 | MT921951 | I | MT920070 | A |
|  |  | R148 | MT921952 | I | MT920071 | A |
|  |  | R149 | MT921953 | VI | MT920072 | A |
|  |  | R150 | MT921954 | I | MT920073 | A |
|  |  | R151 | MT921955 | VII | MT920074 | A |
| Buško Lake,  Bosnia and Herzegovina | Machordom & Doadrio, 2001 |  | AF287416 | II | - | - |
|  |  |  | AF287415 | III | - | - |
| Krka River,  Croatia | Tsigenopoulos & Berrebi, 2000 |  | AF112133 | V | - | - |
| Livno drainage,  Bosnia and Herzegovina | Geiger et al., 2014 |  | - | - | KJ552465 | C |
| Cetina drainage,  Croatia | Geiger et al., 2014 |  | - | - | KJ552727 | B |
|  |  |  | - | - | KJ552629 | A |
|  |  |  | - | - | KJ552412 | A |
| Total | | | 39 | | 37 | |

| Table S3. Variable positions of *cyt b* mtDNA haplotypes in *Aulopyge huegelii*. | | | | | | | |
| --- | --- | --- | --- | --- | --- | --- | --- |
| Haplotype | 105 | 200 | 583 | 669 | 924 | 996 | 1071 |
| I | A | T | C | C | C | G | A |
| II | G | . | . | T | . | A | . |
| III | G | . | . | . | . | A | . |
| IV | . | C | . | . | T | . | . |
| V | . | . | . | . | T | . | . |
| VI | . | . | T | . | T | . | . |
| VII | . | . | . | . | . | . | G |
| Haplotype I: Šator Lake, Studena River; II: Buško Lake; III: Šator Lake, Buško Lake; IV: Šator Lake; V: Šator Lake, Krka River; VI: Studena River; VII: Studena River | | | | | | | |

| Table S4**.** Raw fixed differences (upper matrix) and uncorrected pairwise sequence distance (“*p*” in %) matrix for *cyt b* mtDNA haplotypes of *Aulopyge huegelii*. | | | | | | | |
| --- | --- | --- | --- | --- | --- | --- | --- |
| Haplotype | I | II | III | IV | V | VI | VII |
| I |  | 3 | 2 | 2 | 1 | 2 | 1 |
| II | 0.28 |  | 1 | 4 | 4 | 4 | 4 |
| III | 0.19 | 0.09 |  | 4 | 3 | 4 | 3 |
| IV | 0.19 | 0.38 | 0.38 |  | 1 | 2 | 3 |
| V | 0.09 | 0.38 | 0.28 | 0.09 |  | 1 | 2 |
| VI | 0.19 | 0.38 | 0.38 | 0.19 | 0.09 |  | 3 |
| VII | 0.09 | 0.38 | 0.28 | 0.28 | 0.19 | 0.28 |  |
| Haplotype I: Šator Lake, Studena River; II: Buško Lake; III: Šator Lake, Buško Lake; IV: Šator Lake; V: Šator Lake, Krka River; VI: Studena River; VII: Studena River | | | | | | | |

| Table S5. Raw fixed differences (lower matrix) and uncorrected pairwise sequence (“*p*” in %) distance matrix for *COI* mtDNA haplotypes of *Aulopyge huegelii*. | | | |
| --- | --- | --- | --- |
| Haplotype | A | B | C |
| A |  | 0.15 | 0.15 |
| B | 1 |  | 0.31 |
| C | 1 | 2 |  |
| Haplotype A: Šator Lake, Studena River, Cetina River; B: Cetina River; C: Livno | | | |

| Table S6. Estimates of genetic diversity based on mitochondrial DNA markers in the populations of the *Aulopyge huegelii*. | | | | | | | |
| --- | --- | --- | --- | --- | --- | --- | --- |
|  | Population | *N_t_* | *N_p_* | θ_K_ | θ_H_ | θ*_S_* | θπ |
| *cyt b* mtDNA |  |  |  |  |  |  |  |
| Bosnia and Herzegovina | Šator Lake | 4 (I, III, IV, V) | 1 (IV) | 1.105 | 1.287 | 1.071 | 0.902 |
|  | Studena River | 3 (I, VI, VII) | 2 (VI, VII) | 0.934 | 1.085 | 0.993 | 1.136 |
|  | Buško Lake | 2 (II, III) | 1 (II) | n.a. | n.a. | 1.000 | 1.000 |
| Croatia | Krka river | 1 (V) | 0 | n.a. | n.a. | n.a. | n.a. |
| *COI* mtDNA |  |  |  |  |  |  |  |
| Bosnia and Herzegovina | Šator Lake | 1 (A) | 0 | 0.000 | 0.000 | 0.000 | 0.000 |
|  | Studena River | 1 (A) | 0 | 0.000 | 0.000 | 0.000 | 0.000 |
|  | Livno | 1 (C) | 1 (C) | n.a. | n.a. | 0.000 | 0.000 |
| Croatia | Cetina River | 2 (A, B) | 1 (B) | 1.414 | 1.518 | 0.667 | 0.667 |
| N, sample size; *N_t_*, total number of haplotypes; *N_p_*, number of private haplotypes; θ estimates obtained from: the observed number of haplotypes (θ_K_, *K*, number of distinct haplotypes), the observed homozygosity θ_H_, the observed number of segregating sites (θ*_S_*, *S* = number of polymorphic sites), and from the mean number of pairwise differences θπ. | | | | | | | |

**References**

Geiger, M.F., Herder, F., Monaghan, M.T., Almada, V., Barbieri, R., Bariche, M., ...& Freyhof, J. (2014). [Spatial heterogeneity in the Mediterranean Biodiversity Hotspot affects barcoding accuracy of its freshwater fishes](http://onlinelibrary.wiley.com/doi/10.1111/1755-0998.12257/full). *Molecular Ecology Resources, 14 (6),* 1210-122.

Machordom, A. & Doadrio, I. (2001). Evolutionary history and speciation modes in the cyprinid genus *Barbus. Proceedings of the Royal Society B,* *268*, 1297-1306.

Tsigenopoulos, C.S. & Berrebi, P. (2000). Molecular phylogeny of North Mediterranean freshwater fauna (genus *Barbus*: Cyprinidae) inferred from cytochrome *b* sequences: biogeographic and systematic implications. *Molecular Phylogenetics and Evolution, 14,* 165–179.
